# An exploratory study on the influence of CT slice spacing on the positioning accuracy of 3D-printed patient-specific instruments designed for a distal radius osteotomy

**DOI:** 10.1186/s13018-026-07374-2

**Published:** 2026-11-11

**Authors:** Emilia Gryska, Peter Axelsson, Katleen Libberecht, Charlotte Stor Swinkels, Anders Björkman

**Affiliations:** 1 Department of Hand Surgery, Sahlgrenska University Hospital, Länsmansgatan 20, 431 30 Mölndal, Sweden; 2 Institute of Clinical Sciences, Sahlgrenska Academy, University of Gothenburg, Göteborgsvägen 31, 431 80 Mölndal, Sweden; 3 Department of Medical Physics and Biomedical Engineering, Sahlgrenska University Hospital, Blå stråket 9A, 413 46 Gothenburg, Sweden

**Keywords:** 3D printing, Distal radius, Osteotomy, Patient-specific instruments, Image resolution

## Abstract

**Background:**

Computed tomography (CT) images with slice spacing up to 3 mm are generally sufficient for accurate 3D reconstructions of patient anatomy. For designing patient-specific instruments, particularly for forearm and wrist osteotomies, higher-resolution imaging (slice spacing of 0.4–1.25 mm) is commonly used. However, it remains unclear whether CT slice spacing meaningfully affects the surface fit of 3D-printed patient-specific instruments. This in-vitro study quantified the impact of CT slice spacing on the positional accuracy of instruments for the distal radius.

**Methods:**

Data from 12 patients were analysed. Each had a high-resolution CT image (0.625 mm slice spacing), from which a reference 3D radius model and a patient-specific instrument were designed. The original images were downsampled to slice spacings of 1.0, 1.25, 1.5, 1.875, and 2.5 mm to generate corresponding radius models and instrument bases. An experienced hand surgeon, blinded to slice spacing, fixated each 3D-printed instrument base onto its reference 3D-printed radius model in a simulated surgery. The assembled constructs were scanned, and the translation and rotation deviations of each lower-resolution instrument position were measured relative to the gold-standard instrument from the original scan.

**Results:**

Mean rotational deviations across all slice spacings remained consistently low (< 1°), with the largest of 0.56° around the Z-axis at 2.5 mm. Translational deviations were similarly small (< 0.2 mm), with the greatest mean deviation of − 0.14 mm along the Z-axis at 1 mm. No systematic trend toward increasing error with decreasing image resolution was observed.

**Conclusion:**

CT slice spacing had a generally small effect on the positional accuracy of 3D-printed patient-specific instruments for the distal radius in vitro. These results indicate that clinically acceptable instrument accuracy may be achieved across a broad range of CT slice spacing commonly used in musculoskeletal imaging, potentially reducing the need for high-resolution protocols in selected clinical scenarios.

## Background

Advances in three-dimensional (3D) virtual surgical planning and the development of patient-specific instruments (PSIs) are transforming the practice of orthopaedics and hand surgery. High-resolution computed tomography (CT) data enable the generation of virtual bone models that allow precise visualization and quantitative analysis of complex skeletal deformities [1, 2]. Using these models, a correction can be virtually planned to restore normal anatomy, and PSIs can be designed and fabricated using 3D printing technologies. These PSIs are engineered to conform exactly to the bone surface in their intended position, translating the virtual plan into intraoperative precision [3, 4]. Consequently, the success of deformity correction using PSIs critically depends on the accuracy of the CT-based reconstruction of the patient’s anatomy from which the virtual models are derived.

According to existing studies, CT images with a slice spacing of up to 3 mm enable accurate 3D reconstruction of patient anatomy for visualization purposes [5]. For designing PSIs, particularly for osteotomies of the forearm and wrist, higher-resolution CT images with slice spacing of 0.4–1.25 mm have been used [6–16]. Whether the CT slice spacing used to create virtual 3D models and PSI design affects the fit of a 3D-printed instrument in a patient’s bone has not been studied.

Previous research has examined how the design of the instrument base (the part of the instrument in contact with the bone surface) influences its docking stability on the bone surface, typically assuming an ideal fit between the instrument and bone surface [17–20]. In clinical reality, however, achieving such a perfect fit is challenging. Factors such as the presence of surrounding soft tissues, the spatial resolution of the CT images used for bone reconstruction, and the inherent manufacturing tolerances of 3D printing can all contribute to deviations from the intended bone contact [21]. Building on this, previous investigations have evaluated PSI positioning errors in relation to instrument base design, generally using a fixed CT slice spacing to generate virtual bone models and corresponding instruments [22, 23]. In contrast, only one study by Gryska et al. [24] examined the influence of CT slice spacing on surface discrepancies between high-resolution reference bone models and PSIs designed from lower-resolution virtual models. The study reported that discrepancies approached clinical acceptability for slice spacing up to 1.5 mm but exceeded it for larger slice spacings. However, because that study relied solely on virtual surface comparison, it could not establish whether these discrepancies translate into clinically meaningful positioning errors once PSIs are physically manufactured and docked onto bone. This limitation is further compounded by the fact that existing instrument-fit assessment methods [17] assume the instrument base surface exactly matches the bone surface in its designed position, an assumption violated at lower CT resolutions. This distinction matters clinically because the correction achieved during surgery depends on the PSI’s position relative to the virtual plan once it is docked to the bone, not merely on how closely the designed instrument surface matches the bone surface in a virtual environment. Several factors that are not captured by virtual surface comparison alone may influence this final position. Manufacturing tolerances inherent to 3D printing can introduce small deviations between the designed and printed instrument surface, although previous studies found that the deviations are relatively small (average distance error of 0.27 mm [15]). Therefore, the impact on the PSI fit on the bone is likely negligible. In addition, PSI placement depends on the correct identification of the intended docking position, seating behaviour during application, and the consistency of manual pressure while the PSI is held in place and fixed with K-wires. Evaluating translational and rotational PSI position after physical docking, therefore, captures the cumulative effect of these clinically relevant factors and provides information that cannot be obtained from the virtual surface deviation alone.

To address this gap, this study aimed to examine how CT slice spacing affects the positional accuracy of 3D-printed PSIs when docked onto a reference bone model. We measured translational and rotational deviations in PSIs created for virtual bone models generated using increasingly thicker CT slices, using high-resolution CT-based PSIs as the reference standard.

## Methods

We used CT images and surgical PSI designs from 12 consecutive patients (11 female and one male, median age 61 years, range 21–73 years) scheduled for a corrective osteotomy of the distal radius at our hospital. We used all the data available from a cohort recruited for another study [24]. The study was performed in accordance with the 1964 Declaration of Helsinki.

### Images

The original 12 CT images were acquired on a *GE Discovery CT750 HD* scanner (GE Healthcare, Milwaukee, Wisconsin, USA) with a pixel size of 0.39 mm x 0.39 mm, slice spacing of 0.625 mm, and a slice thickness of 0.625 mm. Thus, the original images consisted of contiguous, non-overlapping slices. For each original image, five additional images were generated by resampling with slice spacings of 1, 1.25, 1.5, 1.875, and 2.5 mm, maintaining the same pixel size. Resampling of the images was done in Python (v3.10.13) using the *resample_img()* function with bicubic interpolation (*interpolation=”continuous”)* from the *nilearn* library (v0.10.2), following a procedure described by Gryska et al. [24].

### 3D radius models

CT image segmentation of the radius was performed using Mimics software (v. 26.0, Materialise NV, Leuven, Belgium). The original high-resolution images were segmented by an experienced hand surgeon (Author 3). The lower-resolution datasets were segmented by a clinical engineer with expertise in 3D virtual surgical planning (Author 1). 3D models were generated using the Calculate Part function in Mimics, set to Optimal quality. Local manual adjustments of the model contours were performed as needed to ensure accurate alignment of the 3D model outline with the corresponding bone boundaries in each CT slice.

**Fig. 1:**
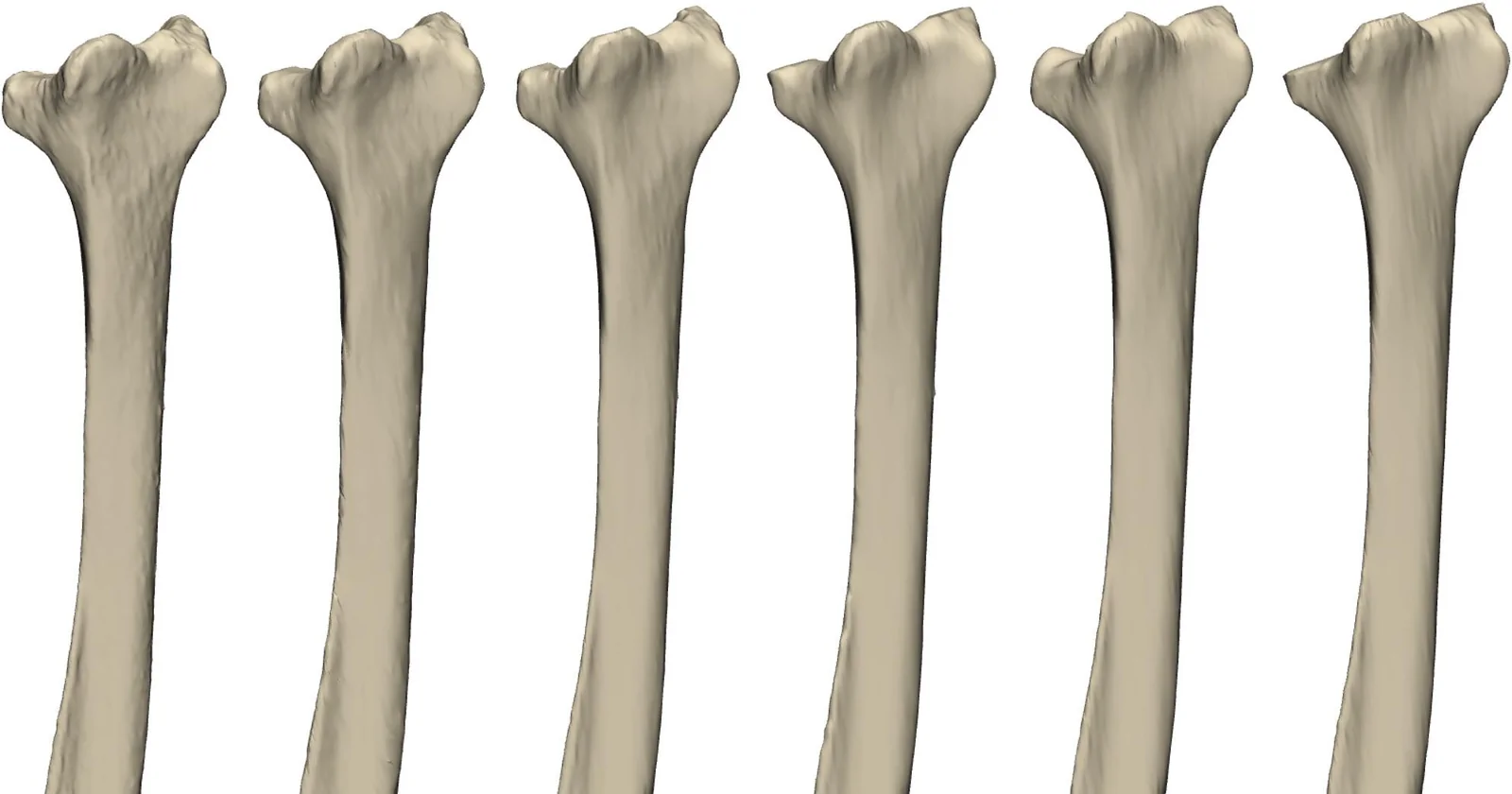
Representative 3D bone models generated from a single patient at slice spacings of (left to right) 0.625-, 1.0-, 1.25-, 1.5-, 1.875-, and 2.5 mm. Increasing slice spacing progressively reduces surface smoothness and fine anatomical detail

As a result, each of the twelve patients had six virtual radius models: one based on the original CT image with a slice spacing of 0.625 mm, and five additional models generated from downsampled CT images with slice spacings of 1.0, 1.25, 1.5, 1.875, and 2.5 mm (Fig. 1).

### Patient-specific instrument base models

**Fig. 2:**
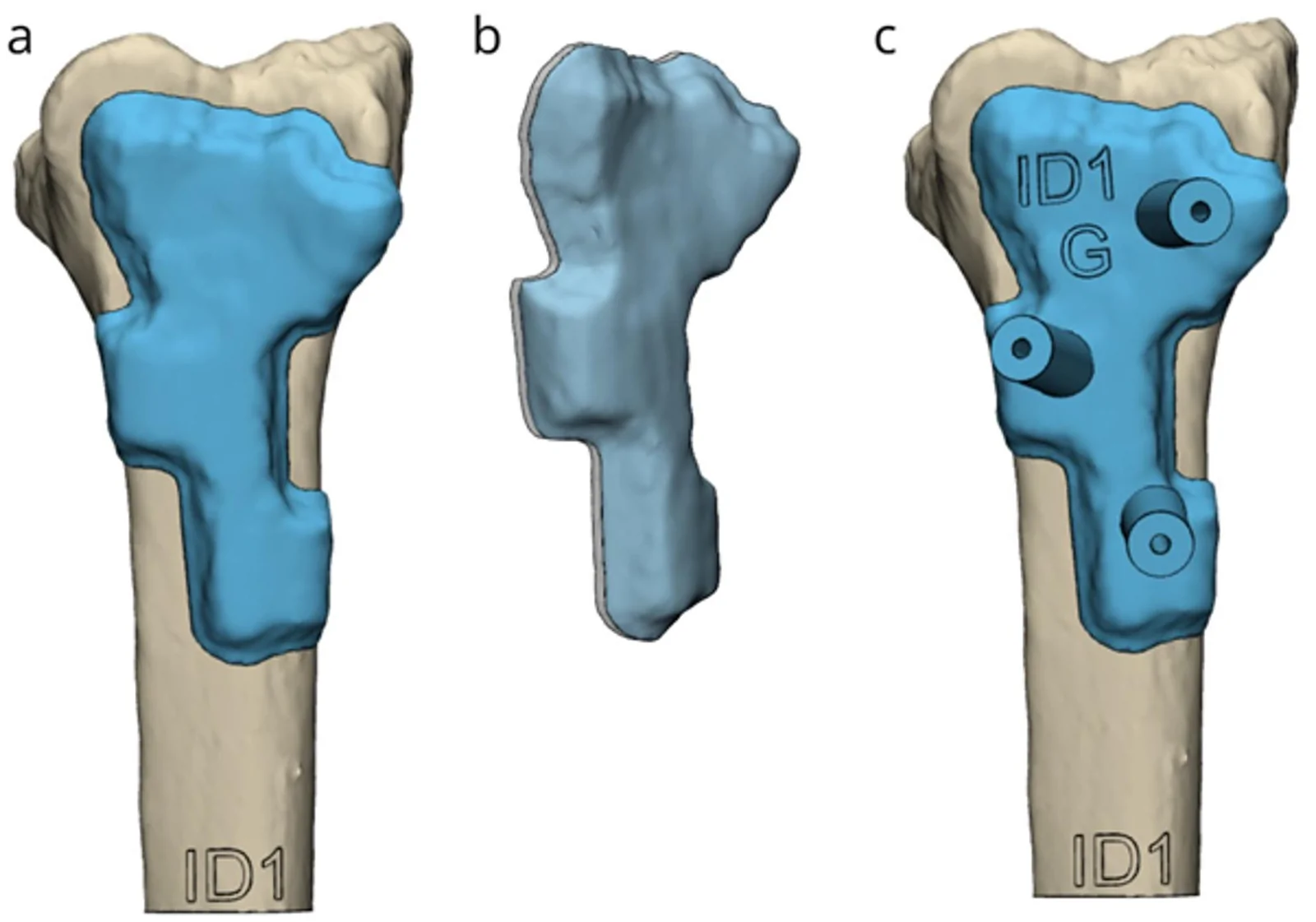
**a** An example of a patient-specific instrument base designed for the original-resolution bone models created from CTs with 0.625 mm slice spacing. **b** The instrument base (blue) was extended by extruding the surface in contact with the bone (grey) to create copies of the same instrument base fitting lower-resolution virtual bone models. **c** A final instrument base with cylinders for fixing the instrument to a bone model

Patient-specific instruments, originally 3D printed for use in surgeries, were designed in 3-matic software (v. 18.0, Materialise NV, Leuven, Belgium) using each patient’s original CT images. The original instrument base, the portion of the instrument that sits flush against the bone (Fig. 2A), and the original virtual radius model were imported from the PSI design file into a new 3-matic instance along with all lower-resolution virtual radius models.

Lower-resolution instrument bases were created by duplicating the original base, extruding the bone-contacting surface (Fig. 2B), and generating five copies of the extruded design. Each copy was fitted to its corresponding lower-resolution radius model by subtracting the bone model from the base with a 0.105 mm clearance, following our established in-house instrument design protocol. Finally, three non-parallel cylinders were added to each instrument base to accommodate 1.5 mm K-wires, which were used to attach the PSIs to the bone (Fig. 2C).

For each patient, six original-resolution radius models were 3D printed, along with the six PSI bases, each fitting the radius model generated from CT images with varying resolution. All models were printed with Bambu Lab X1 in Polylactic Acid (PLA) material at a layer thickness of 0.08 mm.

A hand surgeon experienced in the use of PSIs (Author 2), blinded to the CT slice spacing, fixed each instrument base to its reference radius model in a simulated surgery. This involved positioning the PSI on the bone model and securing it with 1.5 mm K-wires, as during a real osteotomy. The assembled radius–PSI constructs were then scanned using a Revopoint MetroX scanner (Chishine Optoelectronics Technology Co., Ltd., Xi’an, China). Each scanned construct was imported into the 3-matic file containing the virtually designed instrument bases, and the radius portion of each scanned construct was aligned to the reference virtual radius model using the Iterative Closest Point (ICP) algorithm [25].

PSI fit was evaluated by quantifying the positional deviation of the scanned PSIs. For the original-resolution (gold-standard) instruments, a transformation matrix was derived between the scanned instrument position and the virtually planned instrument base position for each patient, establishing the baseline accuracy of the workflow. For the lower-resolution instruments, the transformation matrix was calculated between the scanned instrument position and the scanned position of the corresponding gold-standard instrument, rather than the virtual plan. By quantifying the positional error of the lower-resolution instruments relative to the gold standard, we aimed to isolate CT image resolution as the variable influencing positioning accuracy, while minimizing the impact of other sources of error associated with translating virtually designed instruments into 3D-printed instruments fixed on bone models.

**Fig. 3:**
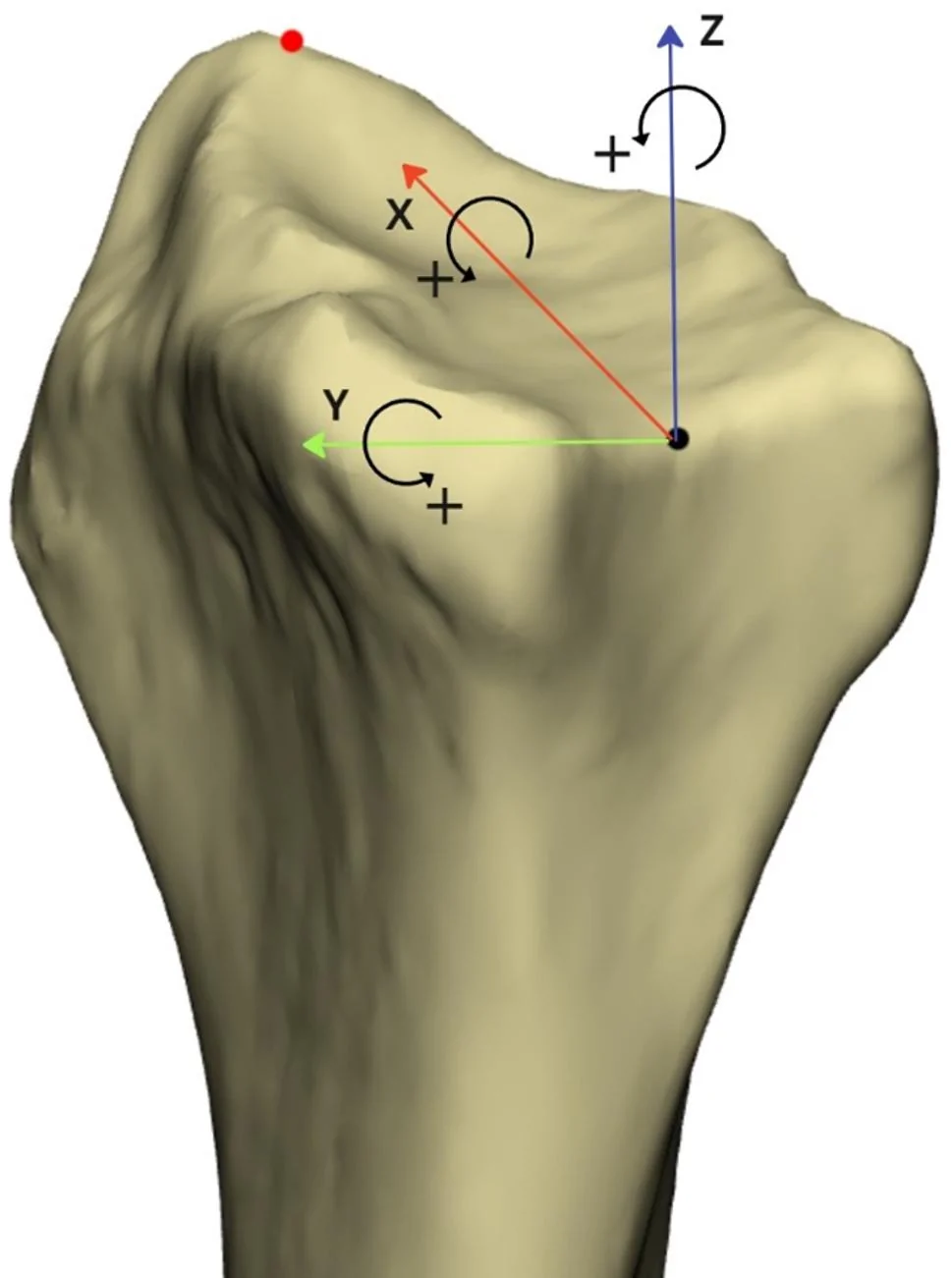
An anatomical coordinate system used to analyse instrument positioning errors. The origin of the coordinate system was defined in the central reference point [26] (black dot). The radial styloid is indicated as a red dot. Figure reprinted from [15] under CC-BY 4.0 license​

The transformation matrix described the translational and rotational deviations along and around the x, y, and z axes of a previously defined 3D anatomical coordinate system (Fig. 3) that was manually identified in each virtual bone model by a hand surgeon (Author 2) [15, 16]. The origin was defined as the centre of the rim between the lunate fossa and sigmoid notch [26]. The z-axis was defined as the central axis of the radial shaft located 3–5 cm proximal to the origin. The x-axis extended from the origin toward the projection of the radial styloid tip, while the y-axis was oriented perpendicular to the XZ-plane. In the anatomical coordinate system, the right-hand-side rotation rules do not apply, so the sign convention is consistent for left- and right-side radii. A positive rotational error around the X-axis indicates PSI rotated relative to its planned position in the direction of increased volar tilt; around the Y-axis: increased radial inclination; and around the Z-axis: increased pronation. Negative values indicate rotation in the opposite direction, i.e., towards dorsal tilt, decreased radial inclination, and supination, respectively. Positive translational errors correspond to the axis directions shown in Fig. 3.

Summary statistics and box plots were generated from the collected data to analyse the effect of image resolution on the fit of the instrument base to the bone. Data from two models (ID7 and ID8), both acquired with 1-mm CT slice spacing, were lost due to errors during scan saving. Because the K-wires used for instrument fixation from the scanned batch had already been removed after scanning to secure the remaining instruments, it was not possible to rescan the missing models.

### Relationship between surface deviation and physical positioning accuracy

To assess whether virtual surface deviation predicted physical instrument positioning accuracy, per-specimen surface deviation values from our previous study, expressed as negative Hausdorff distance [24], were correlated with the corresponding translational and rotational instrument positioning errors measured in the present study. All specimens available in both datasets were included in this analysis (*n* = 58, 60 specimens minus 2 lost scans). Associations were assessed for total translational error, total rotational error, and the individual absolute translational and rotational components along/about the X, Y, and Z axes. Pearson’s correlation coefficient (r), p-values, and coefficients of determination (R²) were used to evaluate the strength of association and the proportion of variance in physical positioning error explained by virtual surface deviation.

## Results

The baseline accuracy of the original resolution representing the gold standard is summarized in Table 1. The mean and standard deviation (SD) of rotational and translational deviations relative to the virtual plan were all below 1° and 0.3 mm, respectively. The largest individual deviations were a rotation of 1.24° around the Z-axis and a translation of 0.39 mm along the Z-axis.

**Table 1:** Mean and standard deviation (SD) of translational and rotational deviations of the original-resolution instruments relative to the virtual surgical plan

Axis	Rotation error [°]			Translation error [mm]		
	X	Y	Z	X	Y	Z
Mean	0.02	− 0.10	0.17	− 0.03	− 0.08	− 0.01
Std	0.20	0.28	0.77	0.16	0.18	0.26

Mean positional deviations of the lower-resolution instruments relative to the original-resolution instruments are presented in Table 2. Across all CT slice spacings, mean rotational errors remained below 1°, with the largest mean deviation of 0.56° around the Z-axis at a slice spacing of 2.5 mm. Mean translational errors were all within 0.2 mm, with the largest mean deviation of − 0.14 mm along the Z-axis, at 1 mm slice spacing.

**Table 2:** Mean and standard deviation (SD) of translational and rotational deviations of the lower-resolution instruments relative to the original-resolution instrument position

Slice spacing [mm]	Rotation error [°]			Translation error [mm]		
	X	Y	Z	X	Y	Z
1	− 0.02 (0.14)	0.07 (0.31)	0.04 (0.46)	0.00 (0.14)	− 0.02 (0.09)	− 0.14 (0.29)
1.25	− 0.06 (0.23)	− 0.08 (0.32)	0.07 (0.65)	− 0.03 (0.1)	− 0.05 (0.14)	− 0.01 (0.27)
1.5	− 0.05 (0.19)	0.10 (0.21)	0.10 (0.65)	0.02 (0.14)	0.00 (0.20)	− 0.02 (0.39)
1.875	− 0.03 (0.28)	0.17 (0.40)	0.12 (0.56)	0.02 (0.14)	0.10 (0.23)	− 0.02 (0.51)
2.5	− 0.07 (0.36)	− 0.01 (0.34)	0.56 (1.55)	0.05 (0.22)	− 0.01 (0.15)	− 0.10 (0.45)

**Fig. 4:**
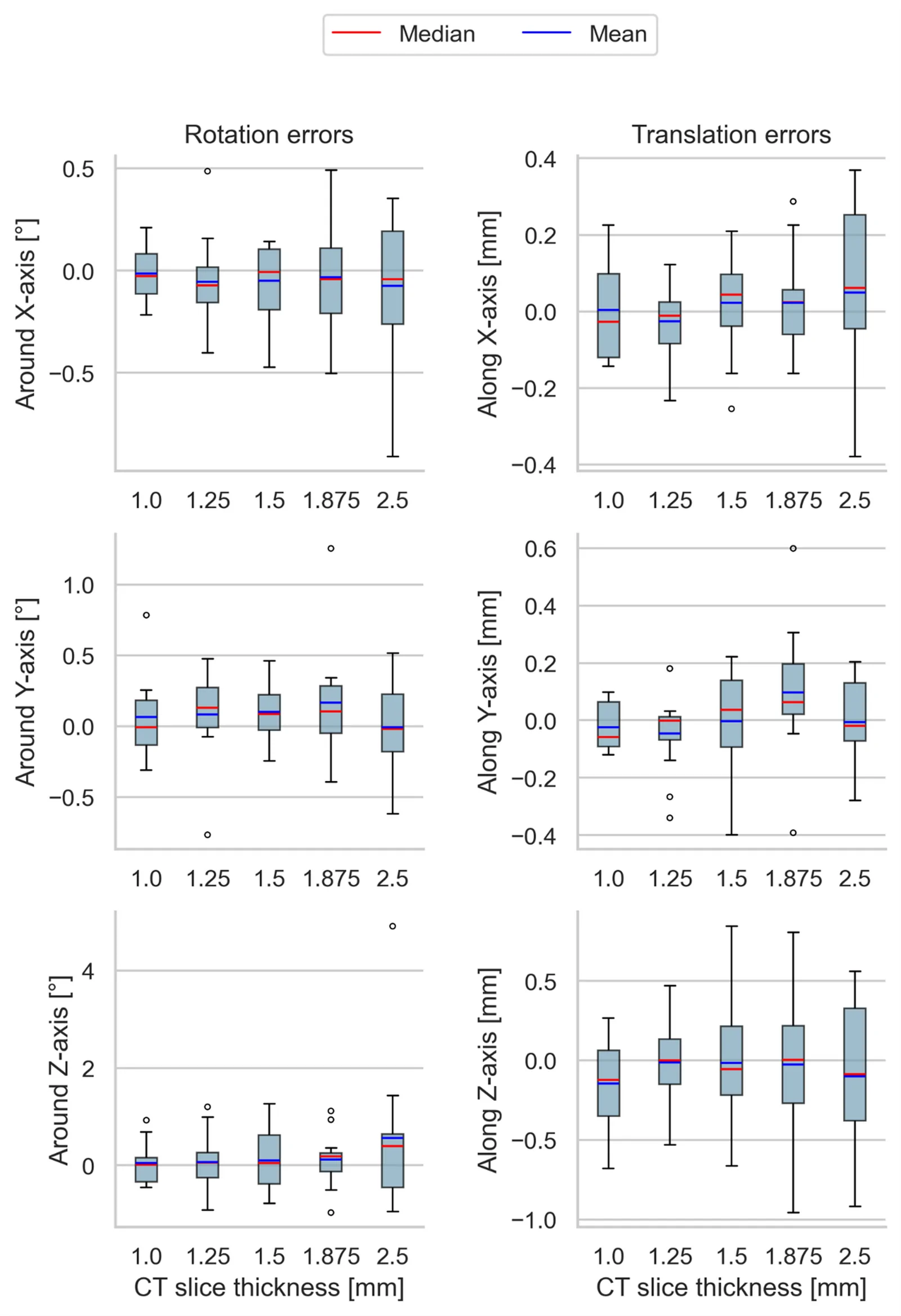
A boxplot of translation and rotation errors of instrument position across the 12 patients, using CT images reconstructed with slice spacings of 1.0, 1.25, 1.5, 1.875, and 2.5 mm. The box shows the quartiles of the data; the whiskers indicate 1.5 times the interquartile range; and the outliers are shown as circles

Although several individual positioning errors of the lower-resolution instruments exceeded the baseline accuracy, all translational errors remained below 1 mm, with most measurements within 0.5 mm (Fig. 4). Rotational deviations were generally within 1°, except for five instances around the Z-axis and one around the Y-axis. The largest individual deviations were observed in instruments designed from CT images with slice spacings of 1.875 mm and 2.5 mm.

**Fig. 5:**
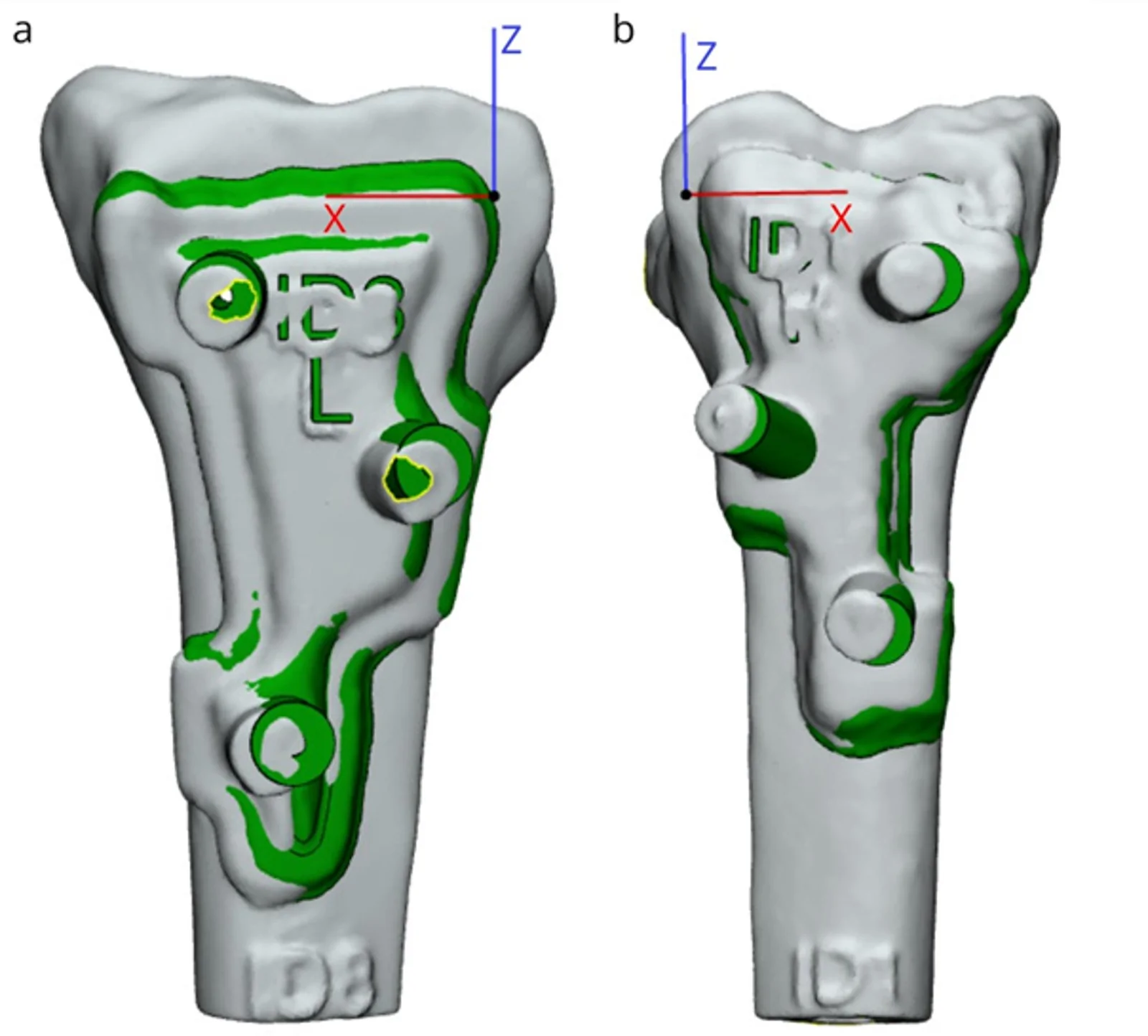
Visualization of the two largest instrument fit errors **a** around the Y-axis with the rotation error of 1.3° for ID8 at 1.875 mm slice spacing, and **b** around the Z-axis with the rotation error of 4.9° for ID1 at 2.5 mm slice spacing

The most pronounced instrument fit error around the Y-axis was observed in model ID8 (1.875 mm slice spacing, rotation error of 1.3°) and around the Z-axis, in model ID1 (2.5 mm slice spacing, rotation error of 4.9°), both shown in Fig. 5.

### Relationship between surface deviation and physical positioning accuracy

**Fig. 6:**
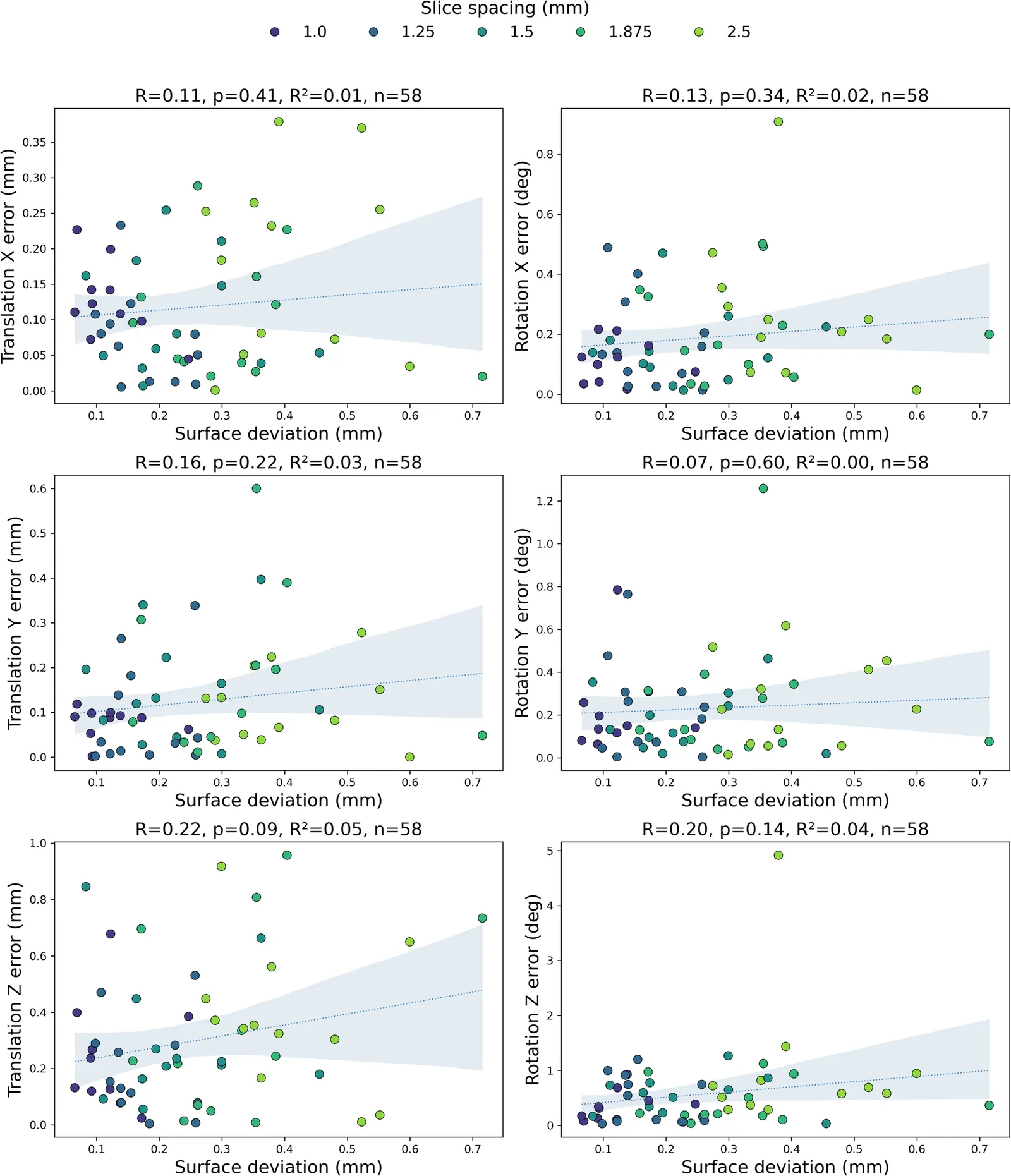
Relationship between virtual surface deviation and physical instrument positioning error. Scatterplots showing the relationship between virtual surface deviation, expressed as negative Hausdorff distance in the previous study [24], and physical instrument positioning error across the absolute translational and rotational components along/about the X, Y, and Z axes. Points are coloured according to slice spacing. The dotted line and shaded band represent the linear regression fit and 95% confidence interval. Pearson’s r, R², and p-values are reported for each panel (*n* = 58). P-values were not corrected for multiple comparisons

Surface deviation showed weak correlation with total translational error (*r* = 0.30, R²=0.09, *p* = 0.02) and no significant correlation with total rotational error (*r* = 0.19, R²=0.04, *p* = 0.14). Among the individual axes (Fig. 6), no significant association was found, and the surface deviation accounted for only up to 5% of the variance in any absolute translational or rotational error component.

## Discussion

This study demonstrated that the examined CT slice spacing range had a negligible impact on the fit of 3D-printed PSIs to 3D-printed bone models with a distal radius malunion. Across all tested resolutions, mean translational and rotational errors remained within 0.2 mm and 1°, respectively, indicating that moderate reductions in CT resolution do not compromise instrument fit accuracy. The largest deviation occurred in rotation around the Z-axis, parallel to the longitudinal axis of the radius. Although the relationship between the instrument position error and correction error following surgery has not been established, we anticipate that the magnitude of errors observed in this study is not large enough to be clinically relevant.

Our results are consistent with previous findings on PSI docking accuracy. Caiti et al. [22] reported similar instrument positioning errors using CT data with a slice spacing comparable to our original-resolution images (0.67 mm). Willemsen et al. [23], who evaluated instruments on cadaveric radii based on CT scans with 0.8 mm slice spacing and 0.4 mm spacing, observed notably larger errors: up to 3.5° and 2.1 mm. This difference likely reflects anatomical and methodological factors: their instruments were positioned on the diaphyseal region, where the absence of distinct bony landmarks reduces rotational constraint, whereas our instruments were designed for the distal radius, which provides well-defined surface morphology for accurate instrument docking.

The original-resolution instrument positioning errors on 3D-printed models were smaller than intraoperatively estimated in Gryska et al.’s clinical study [16]. In surgery, instrument placement is affected by soft tissues, limited visibility, and the difficulty of achieving consistent bone exposure. In contrast, 3D-printed bone models provide idealized conditions, enabling precise scanning and direct comparison of the instrument position to the planned model. Consequently, in-vitro studies such as this one are best suited for systematically evaluating design variables, such as CT resolution. In contrast, clinical studies are crucial for assessing outcomes in real-life settings, where controlling for all sources of error is challenging. It is important to note that 3D-printed models do not perfectly replicate the mechanical and thermal properties of cortical bone. Minor deformations of the printed models can occur when K-wires are inserted, as heat and pressure may slightly distort the plastic model surface. We suspect that this phenomenon contributed to the errors. However, such artifacts are expected to contribute minimally compared to the variability introduced during actual surgical application.

The present findings should be interpreted in relation to our previous study, which assessed the influence of CT slice spacing on instrument accuracy using virtual surface discrepancy between the designed instrument base and the high-resolution reference radius surface. In that study, instruments derived from 1.0 mm slice spacing remained within the clinically acceptable range defined by inter-rater variability, whereas instruments derived from 1.25 mm to 1.5 mm slice spacings approached this limit, and those derived from 1.875 mm to 2.5 mm slice spacings exceeded it. The present study evaluated a downstream endpoint by measuring translational and rotational instrument position after physical docking. The results showed that increased virtual surface discrepancy did not translate directly into a proportional increase in docking error. Instruments derived from slice spacings up to 1.875 mm generally demonstrated mean translational errors below 0.2 mm and rotational errors below 1°, with clinically relevant deviations occurring mainly as isolated outliers rather than as a consistent spacing-dependent trend. Thus, the current study adds clinically relevant information by showing that virtual surface discrepancy alone may not reliably predict the final docked position of a physically manufactured instrument.

To further quantify the relationship between virtual surface deviation and physical instrument positioning accuracy, we correlated per-specimen surface deviation values from our previous study with the corresponding absolute translational and rotational docking errors measured in the present study. Virtual surface deviation showed weak correlation with total translational error (*r* = 0.30, R²=0.09, *p* = 0.02), no significant correlation with total rotational error (*r* = 0.19, R²=0.04, *p* = 0.14), and explained only up to 5% of the variance across all individual translational and rotational components. These findings indicate that virtual surface deviation is, at best, a weak predictor of physical instrument positioning accuracy. Even where the association reached statistical significance, it accounted for less than 10% of the variance in translational error, suggesting that docking accuracy is predominantly determined by other factors. This supports the added value of physical docking validation, because instrument positioning is influenced not only by geometric surface discrepancy but also by factors such as manufacturing tolerances, mechanical seating behaviour, and manual placement. These factors cannot be fully assessed using virtual surface comparison alone. Therefore, physical docking validation provides clinically relevant information about translational and rotational instrument positioning that cannot be inferred reliably from surface deviation analysis alone. By measuring the realized instrument position after physical docking, rather than relying on an assumption of perfect instrument-bone surface congruence, the present study addresses a limitation of previously proposed virtual instrument-fit assessment methods [17]. The current approach provides a physically grounded experimental measure of instrument positioning accuracy and thereby adds information beyond the surface-overlap analysis used in our previous study.

From an imaging and radiation safety perspective, our findings support the use of moderate CT slice spacings (up to approximately 1.25 mm [6–16]) commonly used for 3D virtual surgical planning of corrective osteotomies of the forearm and wrist. Within this range and beyond – up to 1.875 mm – instrument fit accuracy was unaffected, suggesting that thinner slices with the higher radiation exposure they entail [27] may not provide meaningful benefits for PSI design. A clinically relevant deviation of 4.9° was observed only in one instrument, designed from a CT with 2.5 mm slice spacing, which is beyond typical acquisition parameters, particularly for arm and wrist imaging [6–14].

The major limitations of this study were the small sample size and the use of 3D models generated from down-sampled datasets derived from a single original CT acquisition, rather than from CT images independently acquired with different acquisition-level slice thicknesses and slice spacings. Although this resampling approach was more feasible for an exploratory study, the findings should be verified using CT images acquired natively at the tested slice spacings. In summary, the precision of 3D-printed PSIs for the distal radius may be maintained across a clinically practical range of CT resolutions. This finding reinforces the robustness of current CT-based virtual surgical planning and supports efforts to minimize radiation exposure without compromising surgical accuracy.

## Data Availability

Data will be shared upon reasonable request.
